# Food Microbiology: Fundamentals and Frontiers, 5th Edition

**DOI:** 10.3201/eid2801.211862

**Published:** 2022-01

**Authors:** Edward G. Dudley

**Affiliations:** The Pennsylvania State University, University Park, Pennsylvania, USA

**Keywords:** food microbiology, food safety, bacteria

The most recent edition of Food Microbiology: Fundamentals and Frontiers is edited by Michael Doyle, Francisco Diez-Gonzalez, and Colin Hill, well-known names to food microbiologists and possessing broad expertise in pathogenic and beneficial microorganisms (Figure). This book is intended for those with basic knowledge of food microbiology looking for more in-depth discussion of topics commonly covered in such an academic course. Similar to earlier editions, the chapters are divided into 7 sections, beginning with Factors of Special Significance to Food Microbiology, which contains updated chapters on microbial growth in foods, spores, microbiological criteria and indicator organisms, and stress responses. The next section, Microbial Spoilage and Public Health Concerns, details microorganisms found in fresh meats, fruits, vegetables, and nuts. The sections Foodborne Pathogenic Bacteria and Nonbacterial Pathogens and Toxins provide in-depth discussions of many of these agents, including mechanisms of disease, discussions of virulence factors, and host responses. Preservatives and Preservation Methods covers physical, chemical, and biologic methods for control, followed by Fermentations and Beneficial Microbes. The last section, Current Issues and Advances in Food Microbiology, expands on previous editions to include a more complete discussion of genomic and metagenomic applications to food microbiology, providing an overview and discussions of metagenomics in meat and poultry. This expanded final section also includes timely coverage of reclaimed and reconditioned water safety in food production, imported foods, and food safety management systems for ensuring safety across the production chain.

**Figure:**
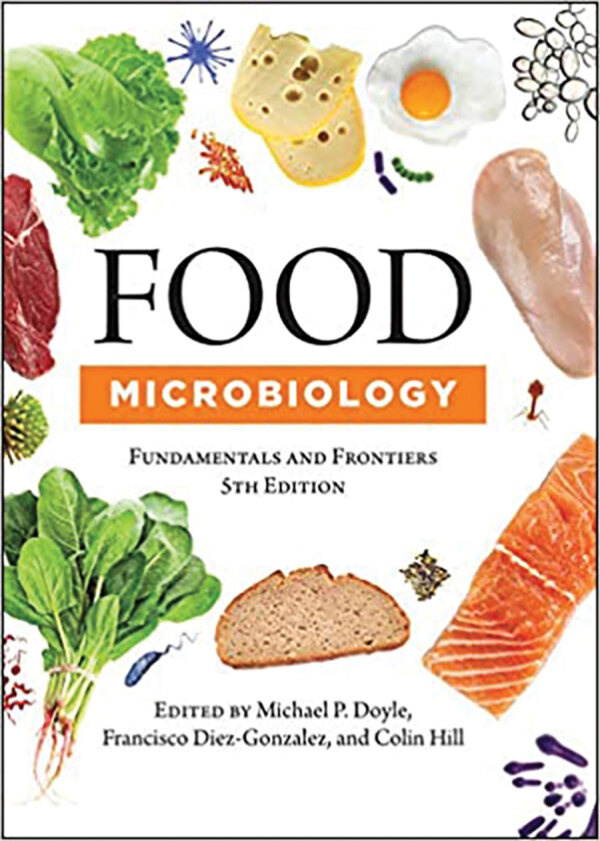
Food Microbiology: Fundamentals and Frontiers, 5th Edition

One notable change is the streamlining of Fermentations and Beneficial Microbes, which historically included chapters on fermented dairy, meat, vegetable products, coffee, cocoa, beer, and wine. These chapters were replaced with Starter Cultures, primarily directed at dairy cultures, and Fermented Foods, which contains limited discussion of the previous topics. The excellent Fermented Foods chapter by Michael Gänzle focuses primarily on fermentations that are specialized to various cultures and includes a reconstruction of his earlier published Periodic Table of Fermented Foods, an amazing tour of 118 products, microorganisms driving each fermentation, and main metabolites and flavor compounds. Another welcome addition is the chapter on bacteriophages for biologic control by Yilmaz Emre Gencay and Lone Brøndsted. Although research has demonstrated challenges in developing practical applications, the use of bacteriophages for pathogen control is of increasing interest. These authors provide extensive tables summarizing previous publications exploring modes of application, experimental parameters, and reductions observed, all excellent resources for experimental scientists in this field.

Future editions could expand on several established and nascent areas. For example, because they are leading agents of foodborne illness, further exploration of specific viruses and their transmission and control in agriculture and food systems, including wastewater systems, is warranted. Although excellent chapters are provided on genomics and source tracking, the recent integration of whole-genome sequencing into PulseNet calls for more discussion, including description of such public resources as the Pathogen Detection program, which currently receives only a mention. Last, although publications are currently limited, emerging areas such as the safety of laboratory-grown meats and food safety in the age of e-commerce would be welcome topics.

